# AI-Mediated Assessment of Continuing Medical Education: The Case-based Learning Intelligence Credit System (CLICS) Framework

**DOI:** 10.2196/99520

**Published:** 2026-09-30

**Authors:** Somkiat Wattanasirichaigoon

**Affiliations:** 1 Center for Continuing Medical Education, Medical Council of Thailand, The Royal Golden Jubilee Building, 2nd Floor Tiwanon Road Talat Khwan, Mueang Nonthaburi District, Nonthaburi, 11000, Thailand, 66 812891665; 2 Dhurakij Pundit University, Bangkok, Thailand; 3 Division of Information Technology Management, Faculty of Engineering, Mahidol University, Nakhon Pathom, Thailand

**Keywords:** continuing medical education, continuing professional development, generative AI, clinical reasoning, medical education, physician learning, large language models, learning analytics, AI assessment

## Abstract

Continuing medical education (CME) and continuing professional development (CPD) systems have traditionally relied on time-based credit allocation, using participation duration as a proxy for professional learning. Although administratively simple and scalable, this model does not reliably demonstrate whether physicians have engaged in meaningful learning, improved clinical reasoning, or critically appraised evidence. The emergence of generative AI creates an opportunity to rethink how physician learning is documented, assessed, and credited. This viewpoint proposes the Case-based Learning Intelligence Credit System (CLICS), a conceptual framework for translating AI-mediated clinical learning interactions into auditable evidence of reasoning-related engagement that could support CME/CPD credit. CLICS introduces the professional learning episode (PLE) as the basic unit of creditable learning: a coherent AI-mediated interaction demonstrating a clinically meaningful problem, reasoning development through iterative inquiry, contextual or evidentiary integration, and reflective synthesis. PLEs are evaluated using the proposed Practice Intelligence Score-7 (PIS-7) rubric, subject to human calibration and oversight; the rubric assesses observable reasoning behavior within the episode rather than the AI’s answer, and qualifying PLEs may be translated into CME/CPD credit through threshold-based, human-auditable conversion rules. CLICS is not intended to replace traditional CME but to extend it as an optional, evidence-generating pathway for personalized, practice-embedded professional development. Its implementation requires iterative validation, stratified human audit, privacy-by-design architecture, antigaming controls, bias monitoring, and professional oversight. If validated, CLICS may enable identification of domain-specific areas for improvement and support personalized, adaptive learning pathways.

## Introduction

Continuing medical education (CME) and continuing professional development (CPD) systems commonly translate attendance, completion, or duration into professional learning credit [1-3]. Although practical and scalable, these participation metrics leave the quality of reasoning and evidence appraisal during learning largely unobserved.

Conversational generative AI creates a new opportunity, because physicians increasingly use it to explore clinical questions, compare alternatives, and reflect on decisions through iterative interactions embedded in clinical practice [4-6].

This viewpoint proposes the Case-based Learning Intelligence Credit System (CLICS), a framework for translating AI-mediated learning traces into auditable CME/CPD evidence. CLICS makes 3 conceptual shifts: from time-based toward evidence-informed CME; from AI as a learning assistant alone toward AI-assisted evaluation of learning traces, subject to human calibration and oversight; and from isolated learning activities toward practice-embedded professional learning infrastructure.

With CLICS, we do not propose that AI independently certify physician competence or that token count, conversation length, or prompt complexity determine credit. Instead, we distinguish superficial AI use from qualifying learning interactions through the professional learning episode (PLE), defined below. Table 1 contrasts conventional CME/CPD with CLICS to show how the proposed framework may complement established systems by recognizing reasoning-centered learning that participation metrics do not capture.

**Table 1.** A conceptual comparison between traditional continuing medical education (CME)/continuing professional development (CPD) and the Case-based Learning Intelligence Credit System (CLICS) framework, showing shifts from participation to reasoning-related evidence.

Dimension	Traditional CME/CPD (established paradigm)	CLICS (proposed paradigm)	Interpretive implication
Foundational assumption	Learning is approximated by time spent in accredited activities	Learning is inferred from observable, reasoning-related engagement within PLEs^a^	Shifts the proxy of learning from duration to observable evidence
Unit of analysis	Educational event (eg, lecture, module, workshop)	PLE (AI-mediated reasoning interaction)	Reframes learning as episodic reasoning rather than attendance-based
Measurement target	Participation and completion	Reasoning-related interaction behavior, inquiry, and reflection	Moves from exposure metrics to reasoning-behavior metrics
Nature of evidence	Indirect (attendance as proxy)	Direct evidence of interaction behavior; inferential evidence of reasoning	Enables partial observation of reasoning-related behaviors
Assessment approach	Often absent or knowledge-based (eg, quizzes)	Multidomain assessment of reasoning-related behavior (PIS-7^b^)	Introduces a structured framework for evaluating reasoning behavior
Temporal context of learning	Scheduled and externally organized	Embedded within real-time clinical problem solving	Aligns CME with practice-based learning moments
Degree of personalization	Limited and content-driven	Potentially high; physician-driven and context-specific	Supports individualized learning trajectories
Feedback structure	Minimal or delayed	Immediate, domain-specific (eg, reasoning depth, critical appraisal)	Enables formative, actionable feedback loops
Auditability	Administrative verification (attendance logs)	Transcript-based audit with human calibration	Expands audit from compliance to reasoning-related evidence
Vulnerability to superficial completion	Possible (passive attendance)	Designed to be mitigated by PLE criteria and reflective synthesis requirements	Introduces structural resistance to low-effort credit accumulation
Governance model	Established regulatory frameworks	Emerging governance (audit, bias control, privacy, AI oversight)	Necessitates new regulatory and ethical architectures
Position within CME ecosystem	Foundational and established	Complementary and exploratory	Supports coexistence rather than replacement

a PLE: professional learning episode.

b PIS-7: Practice Intelligence Score-7.

## Limitations of Time-Based CME

Traditional CME systems have played an important role in supporting lifelong learning, and systematic reviews show that CME can influence physician performance and, in some cases, patient outcomes, particularly when learning is interactive and linked to clinical practice [1,2]. However, CME effectiveness is highly variable and depends on how learning is designed, delivered, and assessed [2,7]. Furthermore, time-based credit allocation has 3 fundamental limitations. First, it relies on participation as a proxy for learning: attendance can be verified administratively, but it does not reflect whether meaningful cognitive engagement has occurred; assessment systems should be grounded in meaningful interpretation of performance rather than exposure time alone [3,8,9]. Second, many CME activities are weakly aligned with real-world clinical reasoning—delivered in structured, preplanned formats that do not reflect the complexity and contextual variability of practice—even though reasoning, defined as the process of collecting information, generating hypotheses, and evaluating evidence, develops through iterative problem-solving and exposure to uncertainty rather than passive information acquisition [10-15]. Third, time-based systems offer limited personalization. Physicians differ in specialty, experience, and practice environment yet typically receive standardized content, even though tailored, context-specific feedback is known to enhance retention and reasoning performance [3,7,15,16].

CLICS addresses these limitations by shifting the focus from learning exposure to learning evidence—asking not how long a physician participated but what observable evidence of reasoning-related engagement is present—consistent with performance-informed assessment principles that emphasize observable evidence of learning rather than participation time alone [8,17].

## The CLICS Framework

CLICS integrates AI-mediated interaction, learning-trace capture, the Practice Intelligence Score-7 (PIS-7), human calibration, and credit conversion within a coordinated CME/CPD infrastructure. Although the underlying CLICS architecture is designed to be applicable across regulated professions requiring CPD, this viewpoint focuses specifically on physician CME, reflecting its current development for physician CME and planned phase 0 feasibility and reliability/agreement evaluation. Profession-specific adaptation, calibration, and governance would be required before extension beyond this scope.

At the foundation of CLICS is the AI-mediated learning trace: the sequence of physician-AI exchanges generated during a problem-centered inquiry [4-6,18-20]. Such traces provide observable, though indirect, evidence of reasoning behavior and must be interpreted cautiously because linguistic output does not fully represent underlying cognition [10,14].

CLICS defines the PLE as its fundamental unit of creditable learning: a coherent, AI-mediated interaction demonstrating (1) a clinically meaningful problem, (2) reasoning development through iterative inquiry, (3) integration of contextual or evidentiary information, and (4) reflective synthesis. Interactions limited to factual lookup, repetitive prompting, or passive copying of AI output do not qualify, a distinction essential to prevent superficial AI use from being misread as meaningful learning.

PLEs are evaluated by an AI-based evaluator that is conceptually distinct from the AI system generating educational content. Its purpose is to assess how the physician engages with the interaction rather than to judge the correctness of the AI’s answer; the PIS-7 rubric used for this assessment is described below [8,9]. Moreover, only qualifying PLEs are eligible for proposed CME/CPD credit. Figure 1 summarizes the closed-loop workflow from inquiry to evaluation, feedback, and subsequent learning.

**Figure 1.**
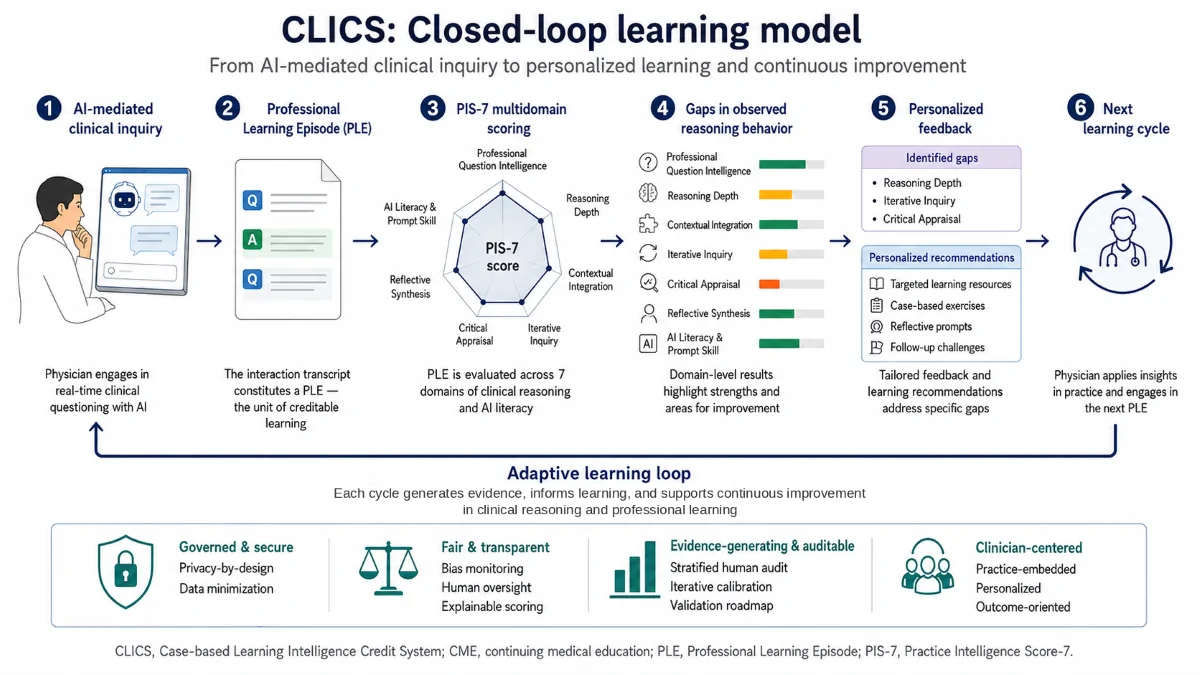
Closed-loop learning model of the Case-based Learning Intelligence Credit System (CLICS). AI-mediated clinical inquiries generate professional learning episodes (PLEs), which are evaluated using the proposed Practice Intelligence Score-7 (PIS-7) framework.

## PLEs and PIS-7 Scoring

PLE components are grounded in the established perspectives that meaningful medical learning involves problem-solving, reasoning, contextualization, and reflection rather than passive exposure [13-15]. Problem framing supports hypothesis generation [10,11]; iterative reasoning supports hypothesis testing and uncertainty management [11,12]; contextual integration incorporates patient, evidence, and system constraints [12,17]; and reflective synthesis supports deeper learning and knowledge transfer [14,15,21]. All 4 components must be present for an interaction to qualify as a PLE.

To operationalize the evaluation of PLEs, CLICS uses the PIS-7 framework, a multidomain rubric designed to assess physician reasoning behavior within a PLE. The 7 domains and their conceptual definitions are summarized in Table 2. Each PIS-7 domain is scored from 0 to 5, and the scores are converted into a weighted composite ranging from 0 to 100:


$$\text{PIS-7}=\sum_{i=1}^{7}\left(\frac{D_{i}}{5}\times W_{i}\right)$$

where Dᵢ is the domain score (0‐5), Wᵢ is the domain weight expressed in percentage points, and ΣWᵢ=100. For example, a score of 4/5 in a 20% domain contributes 16 points.

**Table 2.** Practice Intelligence Score-7 (PIS-7) domains and proposed weighting for evaluating the quality of professional learning episodes.

Domain	Construct assessed	Indicators of high-quality engagement	Weight (%)
Professional question intelligence	Ability to frame clinically meaningful, context-aware questions	Defines relevant clinical problems with appropriate clinical context	15
Reasoning depth	Depth of clinical reasoning and uncertainty management	Demonstrates differential reasoning, prioritization, and risk-benefit analysis	20
Contextual integration	Integration of patient, evidence, and system factors	Incorporates patient factors, guidelines, and local practice constraints	15
Iterative inquiry	Refinement of reasoning through follow-up questions	Uses iterative questioning to clarify uncertainty and explore alternatives	15
Critical appraisal	Evaluation of AI-generated information	Requests evidence, challenges unsupported claims, and recognizes limitations	15
Reflective synthesis	Consolidation of learning into actionable insight	Summarizes learning and articulates implications for clinical practice	10
AI literacy and prompt skill	Responsible and effective use of AI tools	Provides context, verifies outputs, and maintains clinical responsibility	10
Total			100

The weighting scheme assigns the highest weight to reasoning depth (20%), with the other 6 domains weighted at 10% to 15% each. This author-derived asymmetry reflects the conceptual centrality of diagnostic and therapeutic reasoning to physician decision-making [10-12]. The weights have not undergone formal consensus-based elicitation (eg, Delphi) and remain provisional pending empirical validation. After ethics approval and consent, a planned calibration workshop will use synthetic anchor PLEs to train 20 to 25 expert raters on the PIS-7 behavioral anchors before blinded scoring of study PLEs. Human-AI agreement and human interrater reliability will then be assessed using prespecified intraclass correlation coefficient (ICC) analyses to refine the scoring rubric and evaluator configuration rather than to rederive the domain weights.

The composite PIS-7 score summarizes observable reasoning behavior within a PLE rather than physician competence. Outputs should include domain-level explanations, transcript-based justification, and confidence indicators to support transparent human interpretation [8,9]. The PIS-7 is not intended to rank physicians or assign labels of intelligence; its formative purpose is to identify episode-specific areas for improvement and support personalized learning [9,15].

A preliminary credit conversion model may classify PLEs into noncreditable (<40), basic (40–59), meaningful (60–74), advanced (75–89), and very high (≥90) engagement tiers, each associated with a proposed base credit unit of 0, 0.25, 0.5, 0.75, and 1, respectively—consistent with the 0.25‐1 base credit unit range specified in the associated patent claims—with final CPD credit equal to this unit multiplied by a jurisdiction-specific scale factor. Tier labels refer only to engagement within a PLE. These thresholds are conceptual pending empirical calibration; Multimedia Appendix 1 provides 3 illustrative, hypothetical worked examples spanning a nonqualifying interaction and lower- and higher-scoring qualifying PLEs, with domain-level scores, rationale, and proposed credit equivalence. Table 3 summarizes the conceptual workflow from interaction screening to feedback.

**Table 3.** Conceptual screening, scoring, and credit workflow in the Case-based Learning Intelligence Credit System (CLICS).

Stage	Operational question or action	Output
PLE^a^ eligibility gating	Does the interaction demonstrate a clinically meaningful problem, iterative reasoning, contextual or evidentiary integration, and reflective synthesis? Single factual lookups, repetitive prompting, or passive copying do not qualify.	Qualifying PLE, or no PIS-7^b^ score and no credit
PIS-7 scoring	Score 7 reasoning-related domains from 0 to 5 using the proposed weights.	Domain profile and weighted 0 to 100 composite
Blinded human reference and calibration	For phase 0, use a primary set of at least 150 eligible PLEs, with ≥3 independent expert ratings per PLE and AI scores hidden until human scoring is final; if >150 PLEs are available, select 150 by score-stratified random sampling.	Human reference score, human-AI agreement, interrater reliability, and poststudy refinement of scoring anchors
Conceptual credit conversion	Map the composite score to an author-derived engagement tier and base credit unit only as a proposed future conversion model pending empirical validation and regulatory approval.	Illustrative base credit unit (0‐1) × jurisdiction-specific scale factor; no actual continuing medical education/continuing professional development credit in phase 0
Feedback and next cycle	Use domain-level results to identify episode-specific areas for improvement.	Personalized feedback and subsequent PLEs within the closed learning loop

a PLE: professional learning episode.

b PIS-7: Practice Intelligence Score-7.

## Validation Roadmap

A framework that translates AI-mediated learning interactions into CME/CPD credit requires systematic validation of content validity, scoring reliability, construct validity, and implementation validity. Content validity concerns whether the PLE construct and PIS-7 domains adequately represent the intended reasoning-related learning behaviors [8,9]; scoring reliability requires comparison of AI-generated scores with human expert ratings using measures such as the ICC, weighted kappa, and Bland-Altman analysis [22-24]. A recent comparison of ChatGPT (OpenAI) and faculty scoring of formative medical education assessments reported 67% overall exact agreement [25], reinforcing the need for human calibration. Construct validity must establish that higher scores reflect deeper reasoning rather than verbosity [10,11], while implementation validity concerns feasibility, scalability, and auditability.

In the phase 0 pilot study, we plan to enroll up to 75 licensed physicians, each contributing up to 3 PLEs (maximum 225 PLEs), together with an expert-rater pool of 20 to 25 physicians or medical educators. The primary reliability analysis requires a minimum evaluable set of 150 eligible PLEs, each independently scored by at least 3 blinded expert raters; the mean of the assigned human ratings (minimum 3) will serve as the human reference score. Phase 0 is designed to evaluate workflow feasibility and preliminary PIS-7 scoring reliability/agreement; study scores will not be used to award actual CME/CPD credit, affect licensure, or make decisions about physician competence.

If more than 150 eligible PLEs are available, the primary set of 150 will be selected by score-stratified random sampling across low-, middle-, and high-score ranges using locked AI scores before human review; if fewer than 150 are available, feasibility and agreement will be reported as exploratory without claiming validation. Primary human-AI agreement will compare the locked AI composite with the mean human reference using a prespecified absolute agreement ICC with 95% CI, while human interrater reliability will be reported separately; weighted kappa and Bland-Altman analysis will provide secondary agreement checks.

Scoring discrepancies and rubric-clarity feedback will inform subsequent refinement, but the primary evaluator configuration will remain locked throughout the primary analysis. If a critical safety or security change becomes necessary, affected scoring will be paused and the change will be documented under formal change control, with pre- and postchange data separated. These safeguards will preserve the distinction between developmental calibration and post hoc adjustment to observed results.

## Governance, Ethics, and Risk Control

CLICS requires governance for transparency, accountability, fairness, data protection, and human oversight. It should function as an educational assessment system rather than a surveillance mechanism: physicians should know what data are collected and how scores are generated, while expert educators and CME regulatory bodies define thresholds, review audits, and adjudicate disputes [8,9,26-29].

The phase 0 pilot study protocol was submitted to the Central Research Ethics Committee (CREC Thailand), under the supervision of the Foundation for Human Research Promotion in Thailand, on June 24, 2026 (case number CREC(S)24.06.69_01). The CREC reviewed the protocol on August 17, 2026, and requested revision for approval; the revised phase 0 protocol (version 4.1) was resubmitted on August 26, 2026, and remains under review at the time of this revision. The study was prospectively registered with the Thai Clinical Trials Registry on August 1, 2026 (TCTR20260801001), following registry submission on July 23, 2026. No participant or expert-rater scoring data collection has begun, and no such data will be collected before CREC approval is obtained.

For phase 0, research PLEs will use hypothetical or general clinical-learning problems without intentional collection of identifiable patient data, medical records, images, or patient outcomes. Research transcripts will be processed within CCME Version 8.0, the digital infrastructure/platform developed and operated by the Center for Continuing Medical Education (CCME), Medical Council of Thailand, and hosted on Thailand’s Government Data Center and Cloud Service using a prespecified, locally deployed, open-weight, primary PIS-7 evaluator; transcripts will not be transmitted to external commercial AI APIs or used to train or fine-tune external models. Before the first participant PLE, the evaluator model artifact/build, prompt and rubric versions, inference configuration, and relevant run-time environment will be locked and versioned to support reproducibility and auditability.

AI-mediated transcripts may contain patient-adjacent information and indirect identifiers, creating reidentification risk. CLICS should therefore incorporate data minimization, pseudonymization, secure storage, role-based access, and audit logging [27,28], with General Data Protection Regulation (GDPR)–comparable safeguards; under the GDPR, pseudonymized data that remain attributable to an identifiable person are still personal data [30]. Participation should initially be voluntary and restricted to educational use. AI-based evaluators may also be influenced by linguistic style independent of reasoning quality [26]. Generative AI may produce plausible but incorrect information or sycophantically agree with flawed premises [31-35]. Platform-level safeguards are designed to encourage challenges to unsupported premises, explicit uncertainty, and evidence traceability; these measures aim to mitigate, not eliminate, hallucinations and confirmation bias and require empirical validation.

The framework should also include safeguards against gaming, including autonomous or delegated AI agents generating a PLE without the physician’s direct engagement. In any future operational implementation in which qualifying PLEs are converted to CME/CPD credit, reauthentication at the point of credit attribution could strengthen identity assurance. Reauthentication confirms the identity of the individual claiming credit but cannot, by itself, establish that every preceding reasoning step was personally generated by that individual. Additional safeguards may therefore include session-continuity and behavioral-consistency checks, similarity and anomalous-pattern analyses, and credit caps. The reliable technical detection of agent-delegated interactions has not yet been validated; this remains an unresolved governance challenge rather than a solved capability.

## Discussion and Policy Implications

CLICS may encourage a shift from participation-based metrics toward observable evidence of reasoning-related engagement, aligning with broader movements toward performance-informed and evidence-oriented assessment while remaining distinct from formal competency assessment [8,17]. This framing enables domain-specific feedback and individualized learning pathways [14,15,21], while the AI literacy domain reflects the growing integration of AI into clinical workflows [4-6,18-20].

CLICS differs in 3 specific architectural respects from simpler structured reflective CME approaches, such as asking a physician, “What did you learn from this interaction?” First, a standardized Socratic rescue mode, implemented through a response integrity middleware pipeline, can activate when, after 4 physician-AI exchanges, the interaction remains nonqualifying or off-topic. Rather than simply supplying an answer, it returns targeted, nonleading Socratic questions to redirect the interaction toward a qualifying learning process. In phase 0, this mechanism is a standardized learning scaffold rather than an experimental arm, and its activation is analyzed only as an exploratory process metric. Second, once an interaction qualifies as a PLE, the evidence-based response protocol is designed to require traceable literature citations and explicit evidence-level or uncertainty markers for factual claims, providing an auditable evidentiary check that unstructured reflection does not offer. Third, the PIS-7 scores 7 distinct reasoning domains rather than a single undifferentiated judgment, enabling longitudinal, domain-level tracking of reasoning-related behavior and cohort-level analysis—a level of granularity that free-text reflection is not designed to provide.

These structural distinctions provide plausible mechanisms by which CLICS may capture signals unavailable from simpler reflective approaches. This remains a theoretical argument, not a demonstrated finding: CLICS has not yet been shown to be empirically superior to structured reflective CME, and direct comparative evaluation is identified as a priority for future research.

Because AI-mediated learning tools have developed largely outside traditional educational oversight structures, CME/CPD accreditors and regulators are increasingly relevant to quality assurance for AI-mediated learning. This is consistent with guidance from the Accreditation Council for Continuing Medical Education (ACCME), including its January 2026 guidance on responsible AI use in accredited continuing education, its April 2026 alert clarifying that responsibility for learner-facing AI-generated content remains with the accredited provider rather than shifting to a vendor, and the recent literature on governance of generative AI in medical education [36-38]. The response integrity middleware pipeline and evidence-based response protocol function as procedural and evidence-traceability safeguards within a single interaction—enforcing citation and evidence-level marking—but do not by themselves constitute the content-validation infrastructure described in ACCME guidance, which includes predeployment validation against defined clinical scenarios, a defined clinical oversight structure with authority to intervene, ongoing monitoring and revalidation, and vendor accountability sufficient for provider oversight. Commercial bias that is subtle and consistent with evidence is difficult to detect at the level of a single response and is distinct from physician reasoning quality, which is what the PIS-7 measures. Phase 0 intentionally prioritizes reproducibility and research data protection by using a single locked, locally deployed, open-weight, primary evaluator rather than randomizing across external providers. In future operational implementations that incorporate multiple AI providers or knowledge pathways, aggregated PIS-7 and evidence-marker data could support the investigation of systematic models or vendor-specific patterns as 1 supplementary input to independent content validation and accreditor oversight.

A potential strength of CLICS is its alignment with practice-embedded learning: physicians often learn while addressing real problems involving uncertainty [10,13]. CLICS is also designed to mitigate epistemic homogenization by structuring AI-mediated learning around inquiry rather than answer conformity. Beyond the phase 0 evaluation setting, its modular architecture could support multiple models or knowledge pathways to expose learners to alternative interpretations and competing hypotheses; phase 0 intentionally uses a single, locked primary evaluator to maximize reproducibility. In the pilot, the design seeks to support epistemic pluralism through open-ended problems, Socratic counterquestioning, requests for disconfirming evidence, and PIS-7 domains—including iterative inquiry, critical appraisal, contextual integration, and reflective synthesis—which reward reasoned challenge and reflection rather than agreement with a predetermined answer. Repeated PLEs may also provide longitudinal signals relevant to metacognition, including recognition of uncertainty, consideration of alternatives, and self-monitoring of reasoning. These mechanisms aim to support evidence-constrained epistemic pluralism, although their effectiveness in preventing homogenization or improving metacognition remains to be empirically validated.

CLICS has important limitations. Learning traces remain a partial, indirect view of cognition [10,11], and implementation feasibility has not yet been established. The residual evaluator and AI-content risks described above require empirical testing. These limitations support phased validation and cautious interpretation.

From a policy perspective, CLICS is best understood as a complementary pathway that could eventually allow physicians to obtain a portion of CME credit through validated PLEs while continuing established educational activities. Earlier efforts to move beyond time-based participation include the American Nurses Credentialing Center outcome-based continuing education model and competency-based continuing professional development frameworks described in Canada [39,40]. Experience with competency-based assessment in Canada has also highlighted the administrative burden that can accompany frequent assessment and documentation [41]. We do not assume that the use of CLICS will remove such implementation challenges by default. Its plausible points of difference are structural: credit derives directly from a machine-readable transcript rather than a separately authored document, scoring criteria are operationalized as explicit behavioral anchors, and the architecture integrates with AI-mediated learning that physicians already use. Furthermore, because it can engage with novel, nonroutine problems as they arise, CLICS is designed to support CPD at the point of practice in real time rather than only through retrospective reflection on resolved cases. Whether automated capture sufficiently reduces administrative burden and assessor variability to improve sustained adoption remains an empirical question for phase 0 and subsequent phases, not a conclusion asserted here in advance of evidence.

## Conclusions

We propose CLICS, a complementary pathway for recognizing AI-mediated, practice-embedded learning through the PLE and PIS-7. The PIS-7 provides an episode-specific summary of observable reasoning behavior, not a definitive measure of physician competence or clinical performance. If validated, CLICS may support adaptive, evidence-informed professional development by linking feedback and iterative learning to auditable, reasoning-related evidence. Its value will depend on empirical validation, human oversight, and governance addressing content integrity, privacy, fairness, and auditability.

## Supplementary material

10.2196/99520 Multimedia Appendix 1 Illustrative worked examples of Practice Intelligence Score-7 (PIS-7) scoring and continuing medical education (CME) credit conversion.

## Abbreviations

ACCME: Accreditation Council for Continuing Medical Education
CCME: Center for Continuing Medical Education
CLICS: Case-based Learning Intelligence Credit System
CME: continuing medical education
CPD: continuing professional development
ICC: intraclass correlation coefficient
PIS-7: Practice Intelligence Score-7
PLE: professional learning episode
