# Supplementary material for: AI-Mediated Assessment of Continuing Medical Education: The Case-based Learning Intelligence Credit System (CLICS) Framework
Source: JMIR Med Educ. 2026 Sep 30;12:e99520. doi: 10.2196/99520 (PMC13626552; doi:10.2196/99520)
Supplement: Supplementary file 1 [file mededu-v12-e99520-s001.pdf]

Multimedia Appendix 1. Illustrative Worked Examples of PIS-7 Scoring and CME Credit Conversion

Note on interpretation. The three examples below are hypothetical and constructed for illustrative purposes only. They are not transcripts from the Phase 0 pilot study, are not outputs from the locked Phase 0 primary evaluator configuration, and are not part of the planned primary reliability dataset; participant and expert-rater scoring data collection has not begun and remains contingent on CREC approval. The scenarios are deliberately framed at the level of case-based clinical and public health reasoning rather than around specific pharmacologic dosing, laboratory thresholds, or other quantitative clinical facts, consistent with the case-based scope of CLICS across specialties and practice settings. They are provided solely to clarify how the proposed PIS-7 rubric and credit-conversion model could be applied, as requested during peer review. Evidence Level Markers shown in the hypothetical dialogue are illustrative; they do not constitute independently validated clinical guidance. In an operational implementation, the Evidence-Based Response Protocol would additionally require traceable source citations and independent content-validation safeguards.

The composite scores shown below are illustrative and should not be read as fixed or authoritative outputs of the locked Phase 0 evaluator or any other implementation. Phase 0 will use a prespecified, locally deployed open-weight primary evaluator whose model artifact/build, prompt/rubric, inference configuration, and runtime environment are locked before participant data collection; research PLE transcripts will not be transmitted to external commercial AI APIs. AI-generated scores remain research outputs subject to blinded human reference scoring and human oversight.

Table A1. Proposed credit conversion model

Composite PIS-7 score	Tier	Base Credit Unit	Final CPD credit
Fails PLE eligibility criteria	Not scored	—	0
<40	Noncreditable	0	0
40–59	Basic engagement	0.25	0.25 × Scale Factor
60–74	Meaningful engagement	0.50	0.50 × Scale Factor
75–89	Advanced engagement	0.75	0.75 × Scale Factor
≥90	Very high engagement	1.00	1.00 × Scale Factor

Final CPD Credit = Base Credit Unit × Scale Factor, where the Scale Factor is a jurisdiction-specific multiplier set by the relevant professional regulatory body. These thresholds and Base Credit Unit values are provisional and author-derived. They are shown only to illustrate the proposed future conversion logic and will not be applied to participants in Phase 0, which is limited to feasibility and preliminary scoring reliability/agreement.

Example 1 — Non-qualifying interaction (gated before PIS-7 scoring)

Full transcript:

> Physician: What is the usual incubation period for cholera?
>
> AI: The incubation period for cholera is typically a few hours to 5 days, most commonly around 1–2 days. [Grade A]

(No further turns follow.)

Gating outcome: This interaction does not meet the conceptual criteria for a Professional Learning Episode because it is a single factual lookup without iterative reasoning, contextual integration, critical appraisal, or reflective synthesis. CLICS therefore treats it as non-qualifying and does not apply PIS-7 scoring.

Domain scores: Not applicable (gated prior to scoring)

Composite PIS-7 score: Not applicable

Proposed CME credit: 0

Rationale: This example illustrates that CLICS excludes superficial AI use — such as a single factual lookup — through structural eligibility criteria applied before scoring, rather than by assigning it a low PIS-7 score. This directly addresses the concern that credit could be claimed for trivial AI interactions, and applies equally regardless of specialty or practice setting.

Example 2 — Qualifying PLE, meaningful engagement tier

Illustrative scenario: A physician managing a village health post notices an increase in reported acute diarrheal illness over the past week and consults AI to reason through possible explanations and next steps. The exchange comprises four physician–AI exchange cycles followed by a closing physician reflection. The physician provides some local context and requests supporting evidence once. Follow-up questioning is present but limited; critical appraisal of the AI's response is brief, and the closing reflective statement is present but underdeveloped.

Full transcript:

> Physician (turn 1): I'm covering a village health post and have seen more cases of acute watery diarrhea than usual over the past week, mostly in adults from a few neighboring households. What should I be considering?
>
> AI (turn 1): A cluster of acute watery diarrhea across neighboring households raises concern for a common-source exposure, such as contaminated drinking water or food, and warrants basic outbreak investigation — case counts by household and date of onset, a look at shared water or food sources, and consideration of stool testing for common pathogens if feasible. [Grade B]
>
> Physician (turn 2): Is there a general public-health definition or guidance I should use to decide whether this counts as an outbreak that needs to be reported?
>
> AI (turn 2): Public health surveillance systems generally define an outbreak as an increase in cases above what is normally expected for that place and time period, rather than a fixed case count, so comparing the current cluster

against your area's baseline is the key step; local or national reporting thresholds vary by jurisdiction. [Grade B]

Physician (turn 3): The households affected do share a shallow well. Does that change your thinking?

AI (turn 3): A shared shallow well is a plausible common source, particularly for waterborne pathogens, and would support prioritizing water source investigation and safe water messaging while laboratory or environmental confirmation is pending. [Grade B]

Physician (turn 4): Understood — I'll notify the district health office and advise the households on safe water practices in the meantime.

AI (turn 4): That is a reasonable interim step while investigation continues.

Physician (turn 5, closing): Good to know. I'll keep monitoring case counts over the next few days.

Domain scores:

**Domain**	**Score (0–5)**	**Weight (%)**	**Rationale**
D1 Professional Question Intelligence	3	15	Relevant, moderately specific framing of a case cluster
D2 Reasoning Depth	4	20	Reasonable consideration of common-source exposure and next steps
D3 Contextual Integration	3	15	Some local context used (shared well); broader determinants not explored
D4 Iterative Inquiry	3	15	Several follow-up questions are present, but exploration of alternative explanations remains limited
D5 Critical Appraisal	3	15	Seeks a general guidance basis once but does not substantially challenge or independently verify the response
D6 Reflective Synthesis	2	10	Brief closing statement; limited articulation of practice implications
D7 AI Literacy and Prompt Skill	3	10	Adequate context provided; some follow-up refinement

Composite PIS-7 score: 62 (weighted sum 310 ÷ 5)

Tier: Meaningful engagement (60–74)

Proposed CME credit: Base Credit Unit 0.50 × Scale Factor

Rationale: Not all domains score equally — D6 (Reflective Synthesis) is comparatively weak, consistent with the framework's design intent that PIS-7 differentiate strengths and weaknesses within a single PLE rather than require uniformly high performance across all seven domains.

Example 3 — Qualifying PLE, very high engagement tier

Illustrative scenario: A physician reviewing clinic-level surveillance data notices a rise in HIV test positivity over the preceding months and consults AI to reason through the interpretation of this trend. The exchange comprises five physician–AI exchange cycles followed by a closing physician reflection, during which the physician tests competing explanations, explicitly challenges the AI's initial framing and requests graded evidence, integrates local programmatic context, and describes how the exchange will change the physician's approach to interpreting similar surveillance signals in the future.

Full transcript:

Physician (turn 1): At the community clinic I support, the HIV test positivity rate has clearly risen over the past 6 months compared with the year before. What could explain this?

AI (turn 1): A rise in positivity rate could reflect a genuine increase in incidence, an increase in testing coverage or uptake among higher-risk individuals, improved test sensitivity, or some combination of these; a rise in detection alone cannot distinguish a true increase in incidence from a change in who is being tested. [Grade B]

Physician (turn 2): Are you just attributing this to testing bias? I think there may be a genuine increase in transmission in a specific risk group here.

AI (turn 2): I'm not asserting it is only a testing artifact — both explanations are plausible and need to be examined systematically rather than assumed. A useful first step is comparing the trend in testing volume against the trend in positivity rate, since a rising positivity rate that persists after accounting for testing volume is more suggestive of a true increase. [Grade B]

Physician (turn 3): Is there a standard epidemiological approach for telling apart a real increase in incidence from a testing artifact? I want an evidence grade on that, not just a general statement.

AI (turn 3): Public health surveillance practice generally recommends tracking testing volume alongside positivity rate over time and, where possible, triangulating with additional sentinel or behavioral surveillance data rather than relying on positivity rate alone. [Grade B] The specific statistical methods used to adjust for testing-volume changes vary across surveillance systems, and I would not want to imply a single universally validated method exists. [?]

Physician (turn 4): This area recently started outreach testing targeting migrant workers, and there have been informal reports of increased risk behavior locally. How does that factor in?

AI (turn 4): Both factors are plausible contributors. Targeted outreach testing commonly increases detected cases without necessarily reflecting a population-level rise in incidence, while reported increases in risk behavior would be a genuine signal warranting further investigation. Stratifying the data by testing channel — outreach versus routine clinic testing — would help separate these two effects. [Grade B]

Physician (turn 5): Then I'll request the positivity data stratified by testing channel before concluding this represents a real increase in transmission.

AI (turn 5): That is a reasonable next step; keep in mind that aggregate clinic-level data has inherent limitations even after stratification, and findings may still need confirmation through dedicated surveillance or a formal outbreak investigation. [Grade B]

Physician (turn 6, closing): This case has changed how I'll approach similar surveillance signals going forward — I won't conclude that a rise in detection reflects a real increase in transmission until I've ruled out changes in testing coverage or case-finding strategy, especially when a new outreach program has started around the same time.

Domain scores:

**Domain**	**Score (0–5)**	**Weight (%)**	**Rationale**
D1 Professional Question Intelligence	5	15	Precise, context-rich framing of a genuinely ambiguous surveillance signal
D2 Reasoning Depth	5	20	Systematic consideration of competing explanations with explicit reasoning about how to distinguish them
D3 Contextual Integration	4	15	Strong incorporation of local programmatic context (outreach testing, reported risk behavior)
D4 Iterative Inquiry	5	15	Sustained, escalating follow-up questioning that refines the interpretation
D5 Critical Appraisal	5	15	Explicitly challenges the AI's initial framing and requests graded evidence
D6 Reflective Synthesis	4	10	Clear articulation of a specific, practice-relevant change in future interpretation of surveillance data
D7 AI Literacy and Prompt Skill	4	10	Effective, iterative prompting; verifies rather than passively accepts output

Composite PIS-7 score: 93 (weighted sum 465 ÷ 5)

Tier: Very high engagement (≥90). This label describes the observed reasoning behavior captured within this PLE and is not a claim about physician competence or expertise.

Proposed CME credit: Base Credit Unit 1.00 × Scale Factor

Rationale: High scores are earned across nearly all domains, with sustained critical engagement (D5) and systematic reasoning about competing explanations (D2) driving the composite score. The physician explicitly requests an evidence grade and challenges the AI's initial framing. The AI also marks uncertainty rather than inventing a specific quantitative method where none is established. In an operational CLICS implementation, the Evidence-Based Response Protocol would additionally require traceable literature citations for factual claims; the present hypothetical transcript is intended to illustrate PIS-7 reasoning behavior and evidence-level marking, not to substitute for content validation. The scenario is deliberately framed as a population-level, case-based reasoning problem rather than an individual-patient pharmacologic decision, illustrating that PIS-7 is intended for reasoning-related behaviors across clinical and public-health settings.
